# Mechanism of herpesvirus UL24 protein regulating viral immune escape and virulence

**DOI:** 10.3389/fmicb.2023.1268429

**Published:** 2023-09-22

**Authors:** Peilin Ruan, Mingshu Wang, Anchun Cheng, Xinxin Zhao, Qiao Yang, Ying Wu, Shaqiu Zhang, Bin Tian, Juan Huang, Xumin Ou, Qun Gao, Di Sun, Yu He, Zhen Wu, Dekang Zhu, Renyong Jia, Shun Chen, Mafeng Liu

**Affiliations:** ^1^Engineering Research Center of Southwest Animal Disease Prevention and Control Technology, Ministry of Education of the People’s Republic of China, Chengdu, China; ^2^Key Laboratory of Animal Disease and Human Health of Sichuan Province, Chengdu, China; ^3^International Joint Research Center for Animal Disease Prevention and Control of Sichuan Province, Chengdu, China; ^4^Institute of Veterinary Medicine and Immunology, Sichuan Agricultural University, Chengdu, China; ^5^Research Center of Avian Disease, College of Veterinary Medicine, Sichuan Agricultural University, Chengdu, China

**Keywords:** herpesvirus, UL24, immune escape, cGAS-STING, DNA damage response, pathogenicity, virulence

## Abstract

Herpesviruses have evolved a series of abilities involved in the process of host infection that are conducive to virus survival and adaptation to the host, such as immune escape, latent infection, and induction of programmed cell death for sustainable infection. The herpesvirus gene UL24 encodes a highly conserved core protein that plays an important role in effective viral infection. The UL24 protein can inhibit the innate immune response of the host by acting on multiple immune signaling pathways during virus infection, and it also plays a key role in the proliferation and pathogenicity of the virus in the later stage of infection. This article reviews the mechanism by which the UL24 protein mediates herpesvirus immune escape and its effects on viral proliferation and virulence by influencing syncytial formation, DNA damage and the cell cycle. Reviewing these studies will enhance our understanding of the pathogenesis of herpesvirus infection and provide evidence for new strategies to combat against viral infection.

## Introduction

**Figure 1:**
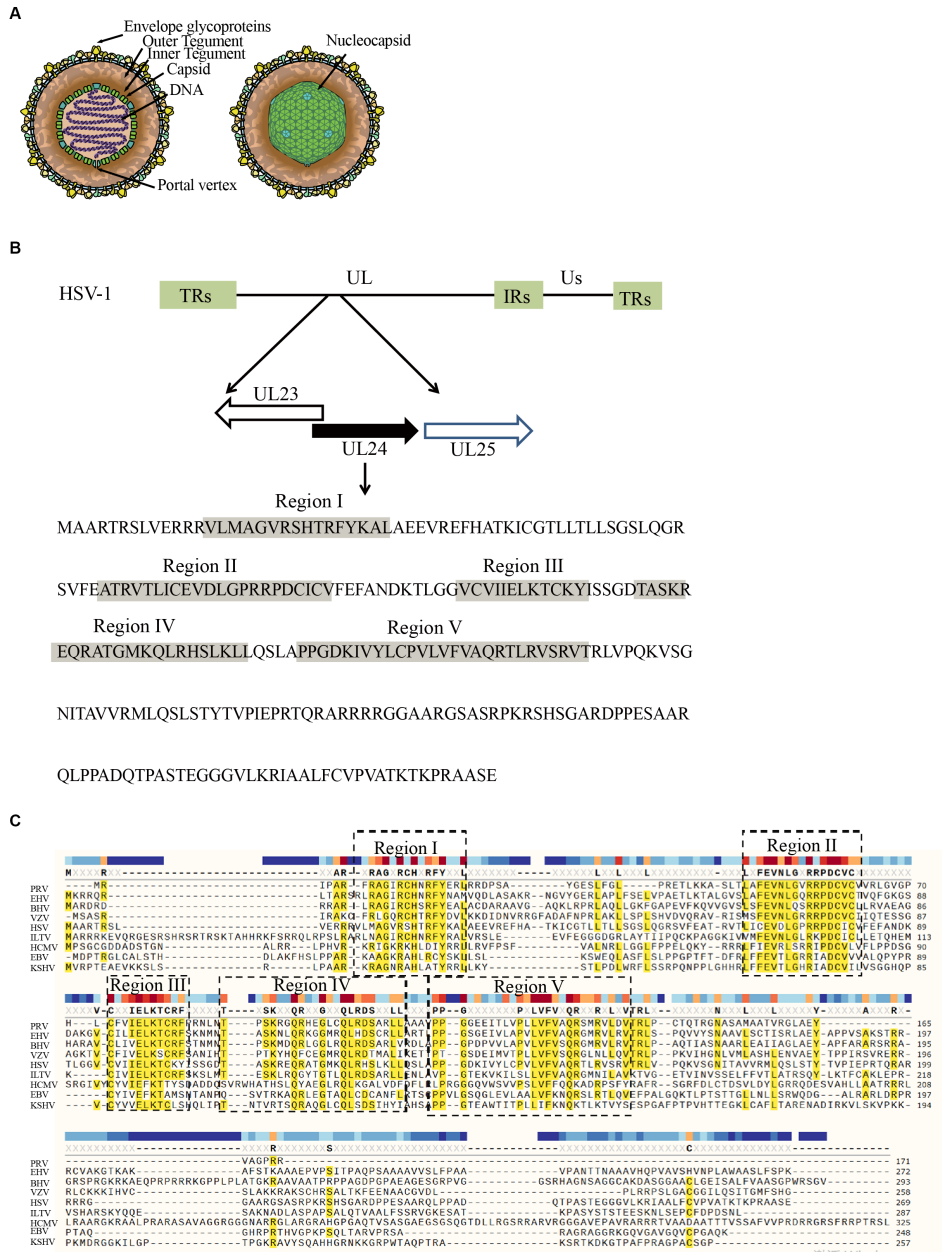
Structure of the HSV-1 genome and the region encoding the UL24 gene. **(A)** Structure of the HSV-1 (Hulo et al., 2011). **(B)** The diagram demonstrates the locations of the UL23, 24, and 25 ORFs and the direction of transcription (arrows) for the parental strain (Pearson and Coen, 2002). Indicated by shading are the locations of domains (Regions I–V) conserved among various herpesvirus homologs of UL24 (Jacobson et al., 1998). **(C)** Multiple alignment of UL24 homologs revealed that they have five conserved functional domains (Dezélée et al., 1996).

Herpesviruses are a group of enveloped, double-stranded DNA viruses with similar biological characteristics that are classified within the Herpesviridae family. To date, more than 100 species have been identified, which are often divided into α, β, and γ subfamilies in addition to unclassified herpesviruses (McGeoch et al., 1995; Boyne and Whitehouse, 2006; Ilouze et al., 2006; Santos, 2016; Rathbun and Szpara, 2021). Herpesvirus possesses a double-stranded DNA genome arranged linearly, enclosed within an icosahedral capsid. Encircling the capsid are tegument proteins, while the outermost layer of the virion consists of a lipid bilayer adorned with proteins and glycoproteins (Figure 1A; Deng et al., 2020; Draganova et al., 2020; Gatherer et al., 2021). Herpesviruses infect the skin, mucous membranes and nervous tissue of a wide range of hosts, seriously affecting the health of humans and other animals (Gupta et al., 2007; Zaravinos et al., 2009; Crimi et al., 2019). Among these, the viruses that often infect humans include herpes simplex virus type 1 and type 2 (HSV-1, HSV-2), varicella zoster virus (VZV), Epstein–Barr virus (EBV), human cytomegalovirus (HCMV), Kaposi’s sarcoma herpes virus (KSHV), and human roseoloviruses, which comprise three different species, human herpesviruses 6A, 6B, and 7 (HHV-6A, HHV-6B, HHV-7), and are genetically related to human cytomegalovirus (Zerboni et al., 2014; Agut et al., 2016; Damania et al., 2022; Ijezie et al., 2023; Martin de Frémont et al., 2023). In addition, horse herpes virus (EHV), pseudorabies virus (PRV), Marek’s disease virus (MDV) and duck plague virus (DPV) infect animals (Pomeranz et al., 2005; Fritsche and Borchers, 2011; Ruan et al., 2022; Zheng et al., 2023). Throughout the host infection process, viral proteins have evolved diverse functions that contribute to the virus’s enhanced survival.

The coding gene of the herpesvirus UL24 protein family is located in a unique long region, and the similarity of amino acids is very high. Except for channel catfish herpesvirus, UL24 protein is conserved in the whole herpesvirus family (Davison, 1992; Bertrand et al., 2010). The proteins encoded by the HCMV UL76, VZV ORF35, KSHV ORF20 and EHV-1 ORF37 genes also belong to the herpesvirus UL24 protein family (Bateman et al., 2004; Ito et al., 2005; Kasem et al., 2010; Hoffman et al., 2021). Except for the HCMV UL76 gene, the UL24 genes and TK genes of other herpesviruses are arranged in a head-to-head manner at the 5′ end (Figure 1B; Jacobson et al., 1989; Dezélée et al., 1996; Shimojima et al., 1997; Pearson and Coen, 2002; Ito et al., 2005; Li et al., 2006).

UL24 is currently considered a core gene of herpesviruses and is present in both mammalian and avian herpesviruses (Nunberg et al., 1989; Lymberopoulos and Pearson, 2007; Jia et al., 2009; Bertrand et al., 2010; Carvalho et al., 2012; Mahmoudian et al., 2012). The transcription of herpesvirus genes presents as a continuous cascade pattern, which is divided into immediate early genes (α), early genes (β), and late genes (γ) according to the chronological order of expression (Sacks et al., 1985; Liu et al., 2015; Zhou et al., 2023). After viral DNA synthesis, UL24 gene products appear in cumulative form late in infection, indicating that UL24 is a late gene (Hong-Yan et al., 2001; Pearson and Coen, 2002; Pearson et al., 2004; Mahmoudian et al., 2012). In herpesviruses, the UL24 protein consists of five highly conserved functional domains that determine most of its functions and are important for the life cycle of the virus (Figure 1C; Jacobson et al., 1989; Shimojima et al., 1997; Knizewski et al., 2006; Nascimento et al., 2009). As herpesvirus research advances, the role of UL24 is becoming increasingly understood. In this review, we examine the function of the UL24 protein by introducing the role of the herpes virus UL24 protein in immune escape, pathogenicity, and the cell cycle.

## UL24 participates in immune escape

**Figure 2:**
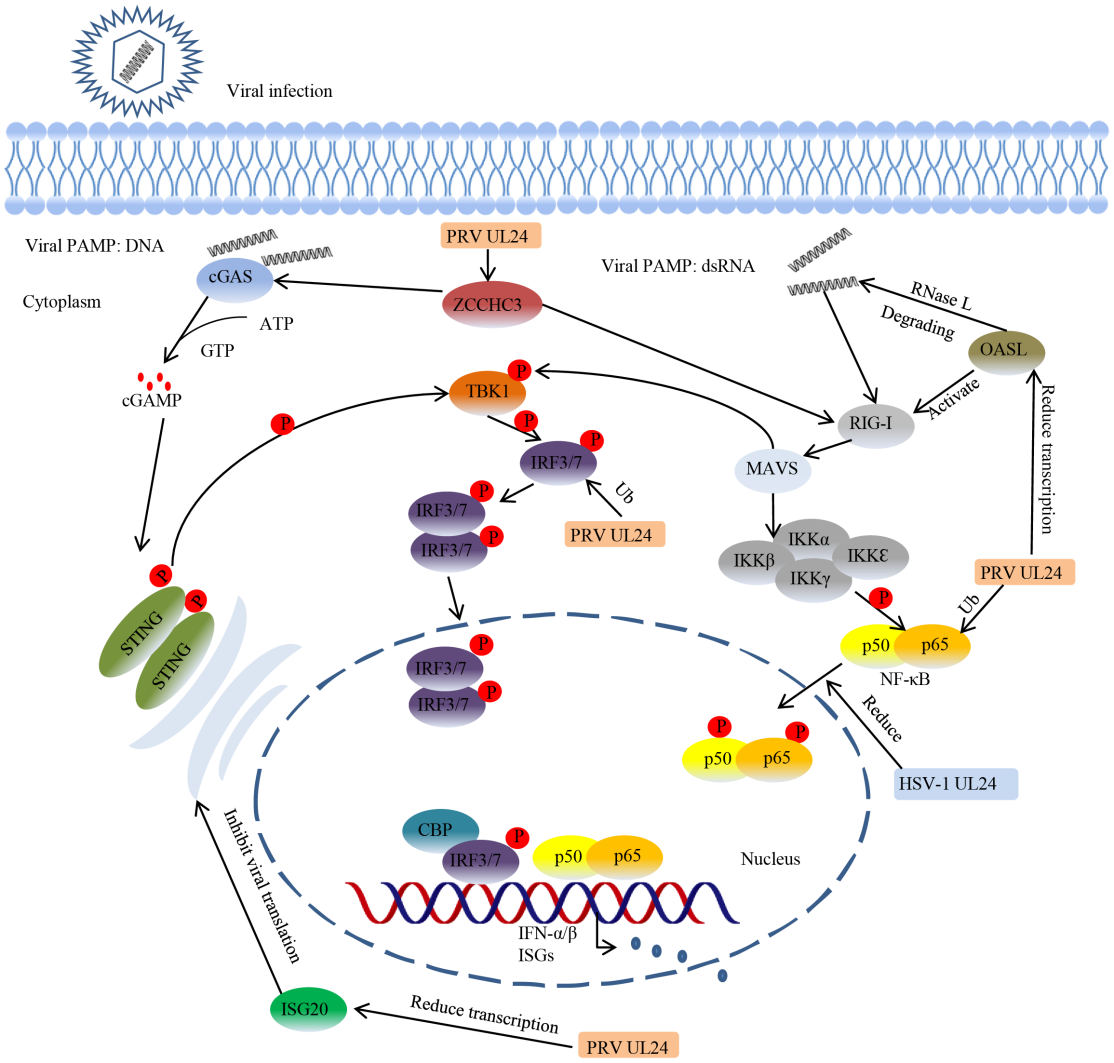
HSV-1 UL24 and PRV UL24 evade innate immunity by inhibiting the cGAS/STING and RIG-I signaling pathways. The host innate immune system can recognize pathogen-associated molecular patterns (PAMPs) through pattern recognition receptors (PRRs), thereby initiating innate immune responses and subsequent adaptive immune responses. Viral PAMPs containing herpesvirus DNA and dsRNA, PRV UL24 and HSV-1 UL24 can inhibit the innate immunity induced by viral PAMPs. PRV UL24 downregulates the expression of the antiviral factors ISG20, OASL and ZCCHZ3 and promotes the degradation of IRF7 and p65 to inhibit the host immune response. HSV-1 UL24 can reduce the entry of NF-κB subunits p50 and p65 into the nucleus to block the signal transmission of the immune pathway.

Innate immune responses are the first line of host defense against pathogens, and host cells recognize pathogens through a series of pattern recognition receptors (PRRs) that trigger the production of type I interferons (IFNs), including IFN-α and IFN-β (Akira et al., 2006; Kawai and Akira, 2010; Liu C. H. et al., 2017; Thoresen et al., 2021; Liu et al., 2022). After binding to the receptors on the cell membrane, IFNs can interact with a series of cellular proteins, eventually leading to the expression of numerous antiviral proteins, thus playing a role in resisting infection and eliminating the virus. To break through this innate immune response and proliferate effectively in host cells, herpesviruses have evolved numerous ways to resist the innate immune response of the host. One of the most important strategies is immune escape (Kolb et al., 2016; Li et al., 2020; Zhu and Zheng, 2020; He et al., 2022; Kong et al., 2022). UL24, as a viral tegument protein, has been shown to act on multiple immune signaling pathways to participate in immune escape from the host antiviral response (Table 1). For example, the HSV-1 UL24 protein interacts with the p65 and p50 subunits of NF-κB and reduces their frequency of nuclear translocation, thereby impeding immune pathway signaling (Xu et al., 2017). The PRV UL24 protein not only blocks the activation of NF-κB induced by tumor necrosis factor-α (TNF-α) by degrading p65 (Wang et al., 2020), but also degrades interferon regulatory factor 7 (IRF7) through the protease pathway to inhibit the cGAS/STING immune pathway and ultimately downregulate the host innate immune response (Liu et al., 2021). Host antiviral factors such as oligoadenylate synthetase-like (OASL), interferon-induced protein 20 (ISG20), and zinc finger CCHC-type containing protein 3 (ZCCHC3) can inhibit the proliferation of herpesvirus (Lian et al., 2018a,b). However, PRV UL24 protein can damage the RIG-I signaling pathway and inhibit the transcription of OASL, interferon-stimulated genes (ISGs) and ZCCHZ3, thus antagonizing the antiviral effects of OASL, ISG20 and ZCCHZ3 (Chen et al., 2021a,b, 2022). Interleukin-8 (IL-8) is a key component of some viruses that infect cells. This cytokine can inhibit the activity of IFN-α and regulate virus transmission and replication (Murayama et al., 1994; Craigen et al., 1997; Khabar et al., 1997). It has been confirmed that HCMV UL76 upregulates IL-8 production (Costa et al., 2013). Therefore, we speculate that HCMV UL76 can upregulate IL-8 and thereby inhibit IFN-α activity, which also has a positive effect on virus resistance to the host immune response. In addition to its role in mammalian herpesviruses, DPV UL24 protein has also been found in avian herpesvirus research to inhibit the activity of IFN-β and participate in immune escape, but the specific mechanism is not clear (Gao et al., 2022). In general, UL24 functions in multiple immune signaling pathways and plays an active role in viral resistance to host immune responses (Figure 2).

**Table 1:** The mechanism by which herpesvirus UL24 participates in immune escape.

Virus	Signaling pathway	Target protein	Mechanism
HSV-1	RIG-I	P65, p50	UL24 inhibits p65 and p50 localization into the nucleus (Xu et al., 2017)
PRV	RIG-I	P65	UL24 induces p65 degradation (ubiquitination) (Wang et al., 2020)
PRV	cGAS-STING	IRF7	UL24 induces IRF7 degradation (ubiquitination) (Liu et al., 2021)
PRV	RIG-I	OASL	UL24 inhibits OASL transcription (Chen et al., 2021a)
PRV	RIG-I	ISG20	UL24 inhibits ISG20 transcription (Chen et al., 2021b)
PRV	RIG-I	ZCCHZ3	UL24 inhibits ZCCHZ3 transcription (Chen et al., 2022)
HCMV	No data	IL-8	UL76 can upregulate IL-8 (Costa et al., 2013)
DPV	cGAS-STING	IFN-β	UL24 inhibits the activity of IFN-β (Gao et al., 2022)

## UL24 affects virus pathogenicity

Viral pathogenicity is usually determined by two factors: the virus itself and host factors. Regarding the herpesvirus, the viral proteins that determine the pathogenicity of the virus are mostly the envelope protein and the tegument protein, which play important roles in the pathogenesis of the herpesvirus (Tang et al., 2017; Shibazaki et al., 2020; Ning et al., 2022; Shen et al., 2023). In a study on the influence of the UL24 protein on virus pathogenicity, it was shown that HSV-1 UL24, especially its conserved domain that influences viral transmission to the host, is important for the virus to cause disease in the host (Leiva-Torres et al., 2010). When mice were infected with a UL24-knockout virus, the transmission of the virus to the trigeminal ganglion was blocked, which greatly reduced the virus titer in the trigeminal ganglion. The mice did not show clinical symptoms, and the latent infection and reactivation of the virus in the trigeminal ganglion were also greatly reduced (Jacobson et al., 1998; Rochette et al., 2015). Reduced pathogenicity was also observed in UL24 mutants of other herpesviruses, such as HSV-2 (Blakeney et al., 2005; Visalli et al., 2014). EHV-1 did not produce any neurotoxicity or lethal effects on mice after deletion of ORF37 (Kasem et al., 2010). The deletion of ORF35 also reduced the pathogenicity of VZV (Ito et al., 2005). In summary, the virulence of herpesviruses was significantly reduced in the existing studies following the deletion of the UL24 gene when compared to the wild-type virus. This indicates that the UL24 protein acts as a virulence factor for the herpesvirus and plays a crucial role in its pathogenicity.

## The role of UL24 in viral replication

A major reason for the reduced virulence of the herpesvirus after the deletion of the UL24 protein is that the proliferation and transmission of the virus are greatly reduced, especially in neurons; this means that UL24 has a regulatory effect on the replication and proliferation of the virus (Rochette et al., 2015). Studies have shown that the UL24 protein is not necessary for the growth of the virus but plays an important role in the replication process of the virus (Ito et al., 2005; Leiva-Torres et al., 2010). For example, HSV-1 replication is downregulated *in vitro* after deletion of UL24 (Sanabria-Solano et al., 2016). During viral infection, OASL usually functions as an antiviral protein that inhibits viral replication and proliferation (Schoggins et al., 2015). However, the expression of OASL during KSHV infection is beneficial to viral replication. Following the deletion of ORF20, the production of new virions and the replication of viral DNA in KSHV-infected cells were significantly reduced compared with those in cells infected with wild-type viruses, suggesting that ORF20 plays a key role in regulating the replication of KSHV (Hoffman et al., 2021). The mechanism involves ORF20 interacting with OASL, leading to an increase in OASL expression and subsequently promoting the replication of KSHV (Bussey et al., 2018). This study is the first to report that KSHV ORF20 can bind with OASL to promote viral replication and proliferation. In addition, zinc finger proteins in host cells play unique biological functions in RNA metabolism, DNA repair and protein processing (Schmitges et al., 2016; Cassandri et al., 2017). Among these, ZCCHZ3 can significantly inhibit the replication of herpesviruses, while the expression of ZCCHZ3 is inhibited when UL24 is overexpressed, which in turn promotes the replication of the virus (Lian et al., 2018a; Chen et al., 2022).

The UL24 protein not only uses host proteins to promote viral replication but also interacts with other viral proteins to participate in the regulation of viral replication. The HSV-1 UL24 protein is also a potential PD-(D/E)XK endonuclease that can interact with a PD-(D/E)XK exonuclease encoded by the UL12 gene to promote the cleavage of redundant viral nucleic acids, which is important for viral replication and provides evidence for the involvement of the UL24 protein in viral replication (Bujnicki and Rychlewski, 2001; Knizewski et al., 2006). Due to the large size of the UL24 family of proteins, the specific role of UL24 homologous proteins in viral replication in individual herpesviruses remains to be confirmed. Dunn W et al. first proposed that HCMV UL76 is a viral replication enhancement gene (Dunn et al., 2003; Yu et al., 2003), while Wang S K et al. showed that the HCMV UL76 gene encodes a protein that inhibits HCMV replication (Wang et al., 2004). Later, Isomura H et al. confirmed that UL76 is involved in the regulation of UL77 gene expression. Since UL77 is important for viral replication, the author speculated that UL76 may be important for HCMV replication (Isomura et al., 2010). Although there are different views on the effect of UL76 on viral replication, the UL24 gene is highly conserved in herpesviruses, so we speculate that HCMV UL76 is an important gene for the promotion of viral replication. In conclusion, the UL24 protein not only interacts with viral proteins to promote viral replication but also regulates host proteins to provide favorable conditions for viral replication.

## UL24 induces nucleolin (C23) and nucleophosmin (B23) distribution

Nucleolar proteins are required for effective infection by herpesviruses, and several nucleolar proteins are repositioned during infection (Callé et al., 2008; Sagou et al., 2010; Strang et al., 2010; Greco et al., 2012; Atari et al., 2022). C23, B23 and fibrillarin are multifunctional nucleolar proteins. They can regulate the transcription of RNA polymerase I and contribute to rRNA maturation and ribosome biogenesis (Boisvert et al., 2007; Mongelard and Bouvet, 2007; Rickards et al., 2007; Cong et al., 2012). During herpesvirus infection, some viral proteins play an important role in the distribution of nucleolin and nucleophosmin (Bertrand and Pearson, 2008; López et al., 2008; Lymberopoulos et al., 2011). Late in HSV-1 infection, nucleolins are dispersed throughout the nucleus in a manner dependent on UL24 protein expression (Lymberopoulos and Pearson, 2007). Ectopic expression of the UL24 protein can also specifically induce the distribution of nucleolin, which confirms that its functional region is a conserved N-terminal region of UL24. In addition, the endonuclease encoded by the UL24 gene is very important due to its function in inducing nucleolar protein dispersal (Lymberopoulos and Pearson, 2007; Bertrand and Pearson, 2008; Bertrand et al., 2010).

## UL24 inhibits cell fusion

Cell fusion is an important biological process that plays an important role in the development, growth and immune responses of organisms (Lu and Kang, 2009; Iosilevskii and Podbilewicz, 2021). Herpesvirus entry and exit from host cells is a complex multistep process, and cell fusion is an important method of entry. Virus-induced cell fusion can be promoted or inhibited by different viral proteins (Manservigi et al., 1977; Connolly et al., 2011, 2021). Vesicles from the Golgi apparatus participate in the further assembly of virions and induce membrane fusion to release newly synthesized virions (Wisner and Johnson, 2004; Mingo et al., 2012; Sucharita et al., 2022). Herpesvirus UL24 protein can be localized to the Golgi apparatus, and its C-terminus is necessary for localization. Therefore, some scholars speculate that the C-terminal domain of the UL24 protein is involved in the regulation of membrane fusion in the late stage of viral infection (Bertrand and Pearson, 2008). The conjecture that the UL24 protein regulates cell fusion was confirmed by a study in which it was found that the UL24-knockout mutant of HSV-1 (UL24X) could not express UL24 protein and therefore lost the function of inhibiting cell fusion (Ben Abdeljelil et al., 2013). The UL24 protein regulates the cell fusion process by interacting with the gB, gD, gK and UL20 proteins (Bzik et al., 1984; Baines et al., 1991; Avitabile et al., 2004; Pataki et al., 2022b). When viruses invade cells, glycoproteins gB, gD, gH and gL form complexes to modify cell membrane proteins and induce the formation of syncytia, the classic manifestation of herpesvirus infection (Atanasiu et al., 2010, 2016; Böhm et al., 2016; Pataki et al., 2022a). As a fusion protein, the gB protein promotes the formation of syncytia, while UL24, gK and UL20 inhibit cell fusion (Atanasiu et al., 2010, 2013; Fan et al., 2023). UL24 can change the localization of gB, gD and F-actin to inhibit cell fusion and cause syncytial plaques in infected cells (Avitabile et al., 2004; Bertrand et al., 2010). Although the formation of syncytia contributes to the spread of the virus between cells, why viral proteins such as UL24 and gK inhibit cell fusion and their specific roles in inhibiting cell fusion remain to be explored. We believe that the UL24 protein’s inhibition of cell fusion might serve as a way to partially shield the virus from being eliminated by the host.

## UL24 induces DNA damage in host cells

Herpesvirus infection can specifically induce chromosome damage in host cells (Fortunato et al., 2000; Fortunato and Spector, 2003; Bencherit et al., 2017). For example, HCMV infection of fibroblasts can induce DNA breakage between DFNA7 and DFNA49 on chromosome 1q23.3, and this damage is associated with hearing impairment (Nystad et al., 2008). Therefore, it is very important to explore the mechanism of DNA damage induced by herpesviruses. Studies have shown that the UL24 homologous protein encoded by the HCMV UL76 gene can cause double-strand breaks in host DNA and increase the amount of cH2AX phosphorylation, followed by the appearance of abnormal chromosomes such as micronuclei (Siew et al., 2009). After DNA damage is induced by the UL76 protein, the expression of IL-8 is upregulated, thus promoting viral replication, and this process also facilitates the effective transmission of the virus through neutrophils (Murayama et al., 1994; Craigen et al., 1997; Costa et al., 2013). While the function of the UL24 protein is typically associated with its five conserved functional domains, the region where the HCMV UL76 protein induces DNA damage is located in its nonconserved C-terminus (Zhang et al., 2015). UL76 interacts with the S5a protein of the ubiquitin protease system and exists in the form of aggregates, and their binding promotes the induction of DNA damage by UL76 (Lin et al., 2013). The S5a protein itself can interact with the DNA damage repair proteins hHR23a, hHR23b and XPC to form complexes (Sugasawa et al., 1997; Hiyama et al., 1999; Fujiwara et al., 2004). Whether UL76 can damage the function of the DNA damage repair complex through S5a and thus inhibit the DNA damage repair process remains to be further studied. In conclusion, herpesvirus UL76 protein induces DNA damage in host cells, which is beneficial to its own survival. Although this conclusion is based on HCMV U76, the UL76 protein belongs to the highly conserved UL24 protein family of herpesviruses. Therefore, we speculate that these results may translate to other herpesviruses.

## UL24 causes cell cycle arrest and induces apoptosis

The cell cycle is a biological clock that controls the phases of life of a cell. The cell cycle is a precise regulatory process of intracellular and extracellular signal interactions. The signaling molecules controlling its operation are cyclin and cyclin-dependent protein kinase (Morris and Divita, 1999; Gutiérrez-Escribano and Nurse, 2015; Swaffer et al., 2016). At different stages of the cell cycle, different cyclin-CDK complexes drive the stable operation of the cell cycle (Gavet and Pines, 2010; Basu et al., 2022). To date, there has been some progress in the study of herpesvirus regulation of the cell cycle, and relevant studies have shown that the viral UL24 protein can cause cell cycle arrest in G2/M phase and cause apoptosis (Ehmann et al., 2000; Song et al., 2000). The cyclin B complex is an important mediator controlling cell cycle transition from G2 phase to mitosis (Ducommun et al., 1991; Smith and Proud, 2008). The expression of the UL24 proteins of HSV-1, MHV-68, HCMV and KSHV in host cells can hyperphosphorylate the Cdc2 protein and increase the expression of cyclin B, thereby downregulating the activity of the Cdc2/cyclin B complex and eventually causing cell cycle arrest at the G2/M phase (Nascimento and Parkhouse, 2007; Nascimento et al., 2009; Paladino et al., 2014). It is an important characteristic of viruses to adapt to the environment of the cell; herpesviruses affect the regulatory proteins of the cell cycle and thereby control the cell division cycle (Paladino et al., 2014; Trapp-Fragnet et al., 2014; Zhao et al., 2019; Bogdanow et al., 2021; Yockteng-Melgar et al., 2022). According to reports, UL24 proteins of α, β and γ herpesviruses can induce cell cycle arrest, which provides favorable conditions for the virus to actively adapt to the environment of the cell.

## ICP27 and TK contribute to virulence by regulating UL24

The expression of proteins is affected by many factors such as interactions between viral proteins form a complex network and can affect the expression or function of other viral proteins. The proteins can combine into complexes to serve the entire life cycle of the virus (Reynolds et al., 2002; Ryckman and Roller, 2004; Liu et al., 2014; Takeshima et al., 2019; Deng et al., 2022). ICP27 is a conserved immediate early protein of herpesviruses that is involved in gene regulation at different stages of the virus. Concurrently, it can terminate host gene expression at the middle stage of viral infection. Its main mechanism is to inhibit mRNA splicing at the posttranscriptional level and to promote nuclear export of transcription products (Koffa et al., 2001; Fontaine-Rodriguez and Knipe, 2008; Tang et al., 2016). The transcription of UL24 is very complex, and the process produces six transcripts. The expression of UL24 protein is mainly related to the expression of transcripts produced by the first transcription initiation site (5.6 kb, 1.4 kb) (Pearson and Coen, 2002). Studies have shown that ICP27 can regulate the production of UL24 transcripts (Pearson et al., 2004). It has been shown that the UL24 protein interacts with ICP27 (Gao et al., 2017). Some scholars have found that ICP27 expression has no effect on the accumulation of UL24 1.4 kb short fragment transcripts but can regulate the transcription level of 5.6 kb long fragment transcripts (Hann et al., 1998). The expression of UL24 protein was reduced by 70% when ICP27-knockout virus was used to infect cells compared with wild-type virus (Pearson et al., 2004). ICP27 not only regulates the expression of UL24 protein but also regulates its cellular localization. It has been found that ICP27 can promote the transport of UL24 from the nucleus to the cytoplasm during viral infection (Gao et al., 2017). In addition, UL23 is also involved in the regulation of UL24. In the early stage of HSV-1 infection, the decrease in thymidine kinase expression promotes the accumulation of UL24 mRNA, especially the 1.4 kb transcription product, which indicates that the attenuation regulation of UL24 mRNA accumulation requires the participation of thymidine kinase (Cook and Coen, 1996; Cook et al., 1996).

## Summary and prospects

In herpesviruses, the UL24 protein, as a component of the tegument, plays a vital role in viral infection of the host. Recent studies have shown that UL24 can induce nucleolar protein redistribution, inhibit cell fusion, induce host cell DNA damage and block progression of the cell cycle, all of which are undoubtedly infectious strategies that have been evolved by viruses for improved survival. In addition, in the process of fighting against the immune response of the host, UL24 also provides great help for the virus to evade the immune response. It can interact with a variety of immune regulatory proteins and antiviral factors to downregulate their expression or inhibit their function and ultimately inhibit the host antiviral response.

The synthesis of new virions in cells is a complex process. There are many studies on the function of the UL24 protein (Leuzinger et al., 2005; Mettenleiter et al., 2006; Sugimoto et al., 2008; Fan et al., 2020), but the specific role of pUL24 in the virus life cycle needs to be further explored.

During primary infection, herpesviruses can establish a lifelong latent infection in the trigeminal ganglion and the pharyngeal tonsil (Doll et al., 2019; Toomer et al., 2022). Although no studies have reported the direct relationship between UL24 protein and latent infection, deletion of UL24 protein can reduce the transmission efficiency of the virus *in vivo* and *in vitro*, especially transmission to the trigeminal ganglion, which may lead to impairment of the establishment and activation of latent viral infection. At present, the research and development of live vaccines and DNA vaccines that use gene deletion is in a rapid development stage, and research on herpesvirus-related vaccines such as PRV, MDV and DPV is relatively mature (Yu et al., 2012; Liu S. A. et al., 2017; Ruan et al., 2022; Sun A. et al., 2022; Sun Y. et al., 2022; Wu et al., 2022; Jiang et al., 2023). Deletion of the UL24 protein can reduce the virulence of the virus, so it is also important to further explore whether UL24-knockout strains can be used as gene deletion candidate vaccines.

The deepening of the understanding of viral proteins will inject new vitality into the treatment of herpesviruses and the development of new vaccines.

## Author contributions

PR: Data curation, Writing – original draft, Writing – review & editing. MW: Conceptualization, Writing – review & editing. AC: Funding acquisition, Project administration, Writing – review & editing. XZ: Writing – review & editing. QY: Writing – review & editing. YW: Writing – review & editing. SZ: Writing – review & editing. BT: Writing – review & editing. JH: Writing – review & editing. XO: Writing – review & editing. QG: Writing – review & editing. DS: Writing – review & editing. YH: Writing – review & editing. ZW: Writing – review & editing. DZ: Writing – review & editing. RJ: Writing – review & editing. SC: Writing – review & editing. ML: Writing – review & editing.

## Funding

The authors declare financial support was received for the research, authorship, and/or publication of this article. This work was supported by China Agriculture Research System of MOF and MARA (CARS-42-17) and the Program Sichuan Veterinary Medicine and Drug Innovation Group of China Agricultural Research System (SCCXTD-2020-18).

## Conflict of interest

The authors declare that the research was conducted in the absence of any commercial or financial relationships that could be construed as a potential conflict of interest.

## Publisher’s note

All claims expressed in this article are solely those of the authors and do not necessarily represent those of their affiliated organizations, or those of the publisher, the editors and the reviewers. Any product that may be evaluated in this article, or claim that may be made by its manufacturer, is not guaranteed or endorsed by the publisher.
